# Supplementary material for: Small Peptides Isolated from Enzymatic Hydrolyzate of Pneumatophorus japonicus Bone Promote Sleep by Regulating Circadian Rhythms
Source: Foods. 2023 Jan 19;12(3):464. doi: 10.3390/foods12030464 (PMC9914586; doi:10.3390/foods12030464)
Supplement: Supplementary file 1 [file foods-12-00464-s001.zip › foods-2143330-supplementary.pdf]

Supplementary material

Method S1: Dextran Gel Sephadex G-15 Separation

The best molecular weight segment for sleep-aiding activity is the <1k Da peptides, so Sephadex G-15 gel was chosen for the separation of the <1k Da peptides.

(1) Preparation of PBPs solution: weighed <1k Da lyophilized PBPs powder, dissolved in distilled water, and prepared into a solution with a concentration of 8 mg/mL. (2) Pre-treatment of dextran gel: The appropriate amount of Sephadex G-15 dextran gel dry powder was weighed at room temperature. in a beaker, add excess distilled water, stir, and leave it to settle naturally, and if there are floating impurities in the upper solution after settling, repeat the step until the last rinsing. If there are impurities such as gel fragments and particles floating in the upper layer of the solution after settling, repeat the step until the last rinse when no suspended matter appears. (This step is to prevent debris particles from blocking the gel column during chromatography and affecting the flow rate). The cleaned gel can be soaked more than soaked in distilled water for at least 24 hours to ensure that the gel is swollen until it is sufficiently swollen, and the volume of the gel no longer changes. The column can be loaded. (3) Loading and equilibration: Fill the bottom of the chromatographic column (1.6×50 cm) with 3-5 cm column height of distilled water. Place the dissolved gel into the column at one time according to the loading requirements, paying attention not to leak out as much as possible, and keep the wet state loading. Avoid bubbles or fractures, which will make the gel density uneven. Due to gravity, when the distance between the settling surface of the gel and the top of the column. The column loading was stopped when the distance between the settling surface of the gel and the top of the column was about 5-8 cm, followed by overnight equilibration of the system with distilled water. (4) Sample loading: Remove the distilled water from the upper layer of the gel column slowly and take 4 mL of *Pneumatophorus japonicus* bone protein peptide solution along the column. The PBPs1 solution was gently injected along the column wall. After the sample solution is infiltrated, add a small amount of distilled water slowly, paying attention to maintain the flat surface of the gel column. Afterward, elute with distilled water at a flow rate of 0.5 mL/min. (5) Elution, detection, and collection: set the UV

detection wavelength to 220 nm, detect the UV absorption peaks, and collect the peaks. The absorption peaks were collected and stored at 4 ℃ for freeze-drying.

Supplementary material

Method S2: Sequence identification of the best active peptide by LC-MS/MS

In peptide sequence determination, LC-MS/MS is usually used to identify amino acid sequence information, which directly determines the advanced structure of a protein or peptide and its active function. Therefore, identifying and analyzing amino acid sequence information of proteins or peptides are essential to investigate their conformational relationships. (1) Sample preparation: 10 mg/mL of collagen peptide solution was prepared by dissolving the lyophilized powder of the best sleep-promoting peptide with distilled water, filtered by 0.22 μm aqueous membrane, and then punched into sample bottles for storage. (2) Protein peptide sequencing was performed with a Q-Exactive Plus mass spectrometer, an EASY-nLC 1200 system, and a C18 column (75 μm×15 cm, 3 μm) for the analysis of PBPs samples. The chromatographic conditions were as follows: mobile phase A: aqueous solution (containing 0.1% formic acid), mobile phase B: 80% acetonitrile solution (containing 0.1% formic acid). The elution gradient was set as follows: 0-3 min, 2%-6%B; 3-42 min, 6%-20%B; 42- 47 min, 22%-35%B; 47-48min, 35%-100%B; 48-60 min, 100%B.

Supplementary material

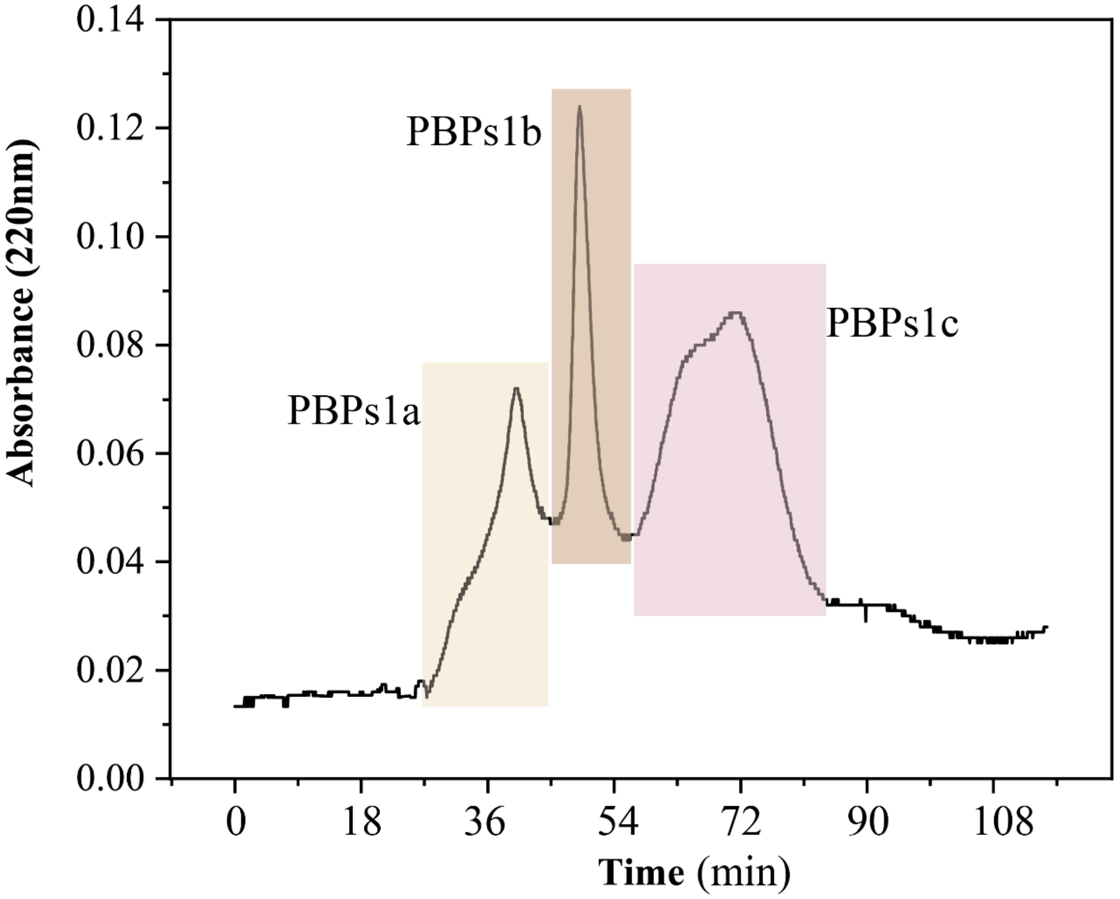

Figure S1. Gel chromatography of PBPs1.

Supplementary material

Table S1 Sequence data generated for the zebrafish transcriptome

Sample name	Raw Reads	Clean reads	clean bases	Error rate (%)	Q20 (%)	Q30 (%)	GC content (%)
ICG-1	42122530	40116770	6.02G	0.02	97.08	94.26	43.78
ICG-2	45194900	42512898	6.38G	0.02	97.97	94.36	44.01
ICG-3	45435116	42057022	6.31G	0.03	96.77	91.56	44.37
MTG-1	42549722	40956028	6.14G	0.03	97.92	94.14	43.52
MTG-2	44901536	42165598	6.32G	0.03	97.86	94.14	44.3
MTG-3	44275970	41996394	6.3G	0.03	97.83	94.03	43.88
TG7-1	41806586	40234444	6.04G	0.03	97.93	94.18	44.09
TG7-2	43226466	41483462	6.22G	0.02	98.01	94.35	42.96
TG7-3	43211824	41621938	6.24G	0.03	97.88	94.15	44.02

Sample: sample name; Raw reads: raw data from the sequencing machine; Clean reads: remaining data after filtering the raw data; Clean bases: the number of clean reads multiplied by the length of sequenced sequences and converted to G units; Q20, Q30: the percentage of bases with base mass fraction greater than 20 and 30 in the total number of bases; GC: the percentage of bases G and C in the total number of bases GC: the sum of bases G and C as a percentage of the total number of bases.

Supplementary material

Table S2 Comparison between reads and reference genome

Sample name	Total_ reads	Total_ map	Multi_ map	Unique_ map	Positive_ map	Negative_ map	Splice_ map	Unsplice_ map
ICG-1	40116770	36508981 (91.01%)	5111893 (12.74%)	31397088 (78.26%)	15679570 (39.08%)	15717518 (39.18%)	8483249 (21.15%)	22913839 (57.12%)
ICG-2	42512898	38591477 (90.78%)	5662287 (13.32%)	32929190 (77.46%)	16445854 (38.68%)	16483336 (38.77%)	9420765 (22.16%)	23508425 (55.3%)
ICG-3	42057022	37236170 (88.54%)	5309007 (12.62%)	31928163 (75.92%)	15938561 (37.9%)	15989602 (38.02%)	9420746 (22.4%)	22507417 (53.52%)
MTG-1	40956028	37103956 (90.59%)	5172998 (12.63%)	31930958 (77.96%)	15951624 (38.95%)	15979334 (39.02%)	8520220 (20.8%)	23410738 (57.16%)
MTG-2	42165598	37808838 (89.67%)	5465967 (12.96%)	32342871 (76.7%)	16150808 (38.3%)	16192063 (38.4%)	9115767 (21.62%)	23227104 (55.09%)
MTG-3	41996394	37758445 (89.91%)	5322064 (12.67%)	32436381 (77.24%)	16192562 (38.56%)	16243819 (38.68%)	9061089 (21.58%)	23375292 (55.66%)
TG7-1	40234444	36695986 (91.21%)	5438838 (13.52%)	31257148 (77.69%)	15610888 (38.8%)	15646260 (38.89%)	8758963 (21.77%)	22498185 (55.92%)
TG7-2	41483462	37561270 (90.55%)	5111907 (12.32%)	32449363 (78.22%)	16202272 (39.06%)	16247091 (39.17%)	8175042 (19.71%)	24274321 (58.52%)
TG7-3	41621938	37501365 (90.1%)	5682874 (13.65%)	31818491 (76.45%)	15886647 (38.17%)	15931844 (38.28%)	8639857 (20.76%)	23178634 (55.69%)

Total map: the number of sequenced sequences that can be localized to the genome; the percentage in parentheses is the percentage of reads in the total clean reads; generally, if there is no contamination and the reference genome is selected appropriately, the percentage of this data is greater than 70%.

Multi map: statistics on the number of sequenced sequences with multiple alignment positions on the reference sequence; the percentage of this part of the data will generally be less than 10%.

Supplementary material

Table S3 Amino acid compositions of PBPs (g/100g)

Amino acid	PBPs1	PBPs2	PBPs3
Asp	0.72 ± 0.01	1.78 ± 0.01	1.25 ± 0.01
Thr	0.43 ± 0.00	0.80 ± 0.01	0.50 ± 0.01
Ser	0.45 ± 0.01	0.78 ± 0.01	0.50 ± 0.01
Glu	0.96 ± 0.01	2.33 ± 0.01	1.59 ± 0.02
Gly	0.72 ± 0.01	1.40 ± 0.01	1.40 ± 0.02
Ala	0.87 ± 0.01	1.44 ± 0.01	1.07 ± 0.13
Val	0.83 ± 0.03	1.10 ± 0.04	0.67 ± 0.02
Met	0.087 ± 0.01	0.15 ± 0.04	0.18 ± 0.24
Lle	0.22 ± 0.01	0.34 ± 0.02	0.21 ± 0.00
Leu	0.65 ± 0.03	0.76 ± 0.04	0.41 ± 0.02
Tyr	0.12 ± 0.01	0.12 ± 0.03	0.04 ± 0.01
Phe	2.18 ± 0.20	2.32 ± 0.37	1.22 ± 0.02
Lys	1.00 ± 0.21	1.08 ± 0.05	0.73 ± 0.05
His	0.26 ± 0.09	0.25 ± 0.06	0.15 ± 0.05
Arg	0.97 ± 0.03	0.91 ± 0.10	0.70 ± 0.47
Pro	0.32 ± 0.20	0.81 ± 0.20	1.01 ± 0.32
Total amino acids	10.79	16.37	11.64

Supplementary material

Table S4 Amino acid sequence of PBPs1c identified by LC-MS/MS

Peptide	-10 lgP	Mass	Length	ppm	m/z	RT	Area Sample
TGVDNPGHPFIK	29.11	1280.651	12	5.1	427.8932	15.54	2.00E+07
EAGPHGPSGPR	28.98	1060.505	11	2.9	531.2613	7.43	3.32E+05
NWPTYPQ	27.47	904.4079	7	5.3	905.42	29.46	1.25E+06
VHHVP	26.49	587.318	5	0	588.3253	42.44	0
DGADFAKWR	26.19	1064.504	9	4.9	533.2618	20.02	4.97E+07
VNIGTIGHVDH	25.26	1160.594	11	3	581.3059	18.22	8.19E+06
VPLQDVYKIG	24.73	1130.634	10	3.3	566.3259	42.91	2.23E+06
SYH	24.69	405.1648	3	3.7	406.1736	36.11	1.11E+07
LDC (+57.02) HTAHIAC (+57.02)	24.36	1196.507	10	4.1	599.2631	10.13	8.32E+05
GC (+57.02) TSVIC (+57.02) SDK	23.87	1125.48	10	1.8	563.748	9.4	1.93E+06
HFNDPVH	23.83	864.3878	7	5.6	433.2036	9.07	1.86E+07
AVDPVYPPGPPAFPK	23.69	1550.813	15	2.9	776.4161	49.32	5.83E+05
NPLFPPGPPK	23.17	1062.586	10	3.2	532.3021	36.32	0
PSGPVGPAGK	23.03	865.4657	10	3.8	433.7418	8.11	1.11E+07
LYDQHLGK	22.1	972.5029	8	2.7	487.26	9.28	1.80E+06
LYDQHIGK	22.1	972.5029	8	2.7	487.26	9.28	1.80E+06
YDQHLGKT	21.62	960.4665	8	5.8	481.2433	7.84	9.96E+05
YDQHIGKT	21.62	960.4665	8	5.8	481.2433	7.84	9.96E+05
HDC (+57.02) WPNC (+57.02) T	21.39	1088.38	8	4.1	545.1997	12.05	8.94E+06
DIVYPPGPPSNPR	21.14	1407.715	13	3.8	704.8672	29.34	1.98E+07

GFAGDDAPR	21.04	904.4039	9	3.4	453.2108	9.53	2.77E+07
VSFPY	20.81	611.2955	5	2	612.304	34.44	1.50E+07
YGNPWEK	20.56	892.4079	7	2.6	447.2124	13.46	1.55E+08
HERDPTQI	20.22	994.4832	8	5.6	498.2516	8.6	2.06E+06
IDK	20.16	374.2165	3	3.6	375.2251	8.47	6.09E+07
LDK	20.16	374.2165	3	3.6	375.2251	8.47	6.09E+07
AGFAGDDAPR	20.08	975.441	10	2.5	488.729	10.32	1.16E+07
TVGF	20.03	422.2165	4	2.1	423.2247	20.87	1.31E+08
